# Proximity Environmental Feature Based Tree Health Assessment Scheme Using Internet of Things and Machine Learning Algorithm

**DOI:** 10.3390/s19143115

**Published:** 2019-07-15

**Authors:** Yang Wei, Hao Wang, Kim Fung Tsang, Yucheng Liu, Chung Kit Wu, Hongxu Zhu, Yuk-Tak Chow, Faan Hei Hung

**Affiliations:** Department of Electrical Engineering, City University of Hong Kong, Hong Kong 999077, China

**Keywords:** tree health assessment, proximity environmental feature (PEF), adaptive data identifying (ADI) algorithm, radial basis function neural network (RBF NN)

## Abstract

Improperly grown trees may cause huge hazards to the environment and to humans, through e.g., climate change, soil erosion, etc. A proximity environmental feature-based tree health assessment (PTA) scheme is proposed to prevent these hazards by providing guidance for early warning methods of potential poor tree health. In PTA development, tree health is defined and evaluated based on proximity environmental features (PEFs). The PEF takes into consideration the seven surrounding ambient features that strongly impact tree health. The PEFs were measured by the deployed smart sensors surrounding trees. A database composed of tree health and relative PEFs was established for further analysis. An adaptive data identifying (ADI) algorithm is applied to exclude the influence of interference factors in the database. Finally, the radial basis function (RBF) neural network (NN), a machine leaning algorithm, has been identified as the appropriate tool with which to correlate tree health and PEFs to establish the PTA algorithm. One of the salient features of PTA is that the algorithm can evaluate, and thus monitor, tree health remotely and automatically from smart sensor data by taking advantage of the well-established internet of things (IoT) network and machine learning algorithm.

## 1. Introduction

As one of the most common species on earth, trees play an important role in protecting the ecological environment, and also have a great impact on human activities. Healthy trees benefit the lives of human beings. Specifically, trees have an effect on soil erosion prevention, carbon dioxide absorption, air purification, noise reduction, dust identifying and landscape design. In contrast, improperly grown trees can cause potential hazards such as soil erosion and global warming. Huge economic loss can also be incurred. For example, it is estimated that the total cost of damage and erosion prevention are 44.4 billion dollars every year [1], which could have been avoided by dedicating more resources to tree health. In essence, trees alleviate global warming by absorbing greenhouse gases. Without healthy trees, global warming will intensify and cause greater harm [2]. For instance, global warming yields dry weather and harms agricultural production. Agricultural crops will not grow well due to high temperatures and droughts. Evidence of this is shown in the fact that a direct economic loss of 5.4 billion dollars was caused in China in 2017 [3].

It is envisaged that an effective tree health evaluation helps to monitor tree health, thus providing early warnings when there is a problem. In general, tree health evaluation is conducted manually by experts according to established standards [4]. In past years, considerable research on tree health assessments has been undertaken. These methods were successfully applied widely and have achieved good performance [5,6]. However, the disadvantage of traditional evaluation methods is that the time and frequency of tree health assessments is limited, and a huge amount of labor by tree experts is required. In other words, they are inefficient. As the technologies developed, various assessment methods based on satellite data and hyperspectral image have been proposed as effective solutions. Remote sensing-based tree health assessment algorithms are a novel method which relies on the remote sensing images captured by remote sensing satellite. In general, information derived from satellite data were utilized for forest and urban tree studies [7,8,9]. These studies included Normalized Difference Vegetation Index-based [10] vegetation abundance and cover estimation [11,12], infrared imagery-based urban tree health mapping [13], and Leaf Area Index-based tree health estimation [14]. The approach is effective but there are still some problems. In scenarios in which there are a large number of objects, the performance will be influenced because of coarse multispectral data (e.g., it is hard to distinguish items in a scenario with a high abundance of mixed pixels). The presence of background materials within a pixel negatively affects the reliability of the sensing data [15]. In addition, since such images present a bird’s eye view, the health conditions of other parts of the tree that are obscured by the leaves cannot be detected.

Recent developments in hyperspectral remote sensing technologies provide another new solution for tree health mapping. Hyperspectral imaged-based tree health assessments rely on the analysis of hyperspectral images captured by hyperspectral cameras [16]. This has become a more popular tool for high-throughput tree phenotyping, because it provides remarkable insights into tree health incorporating non-invasive sensors, computer vision, as well as data mining techniques. Hyperspectral imaging has been shown to be able to reveal the physiological and structural characteristics of trees and to track physiological dynamics under environmental influences [17]. Through comparing hyperspectral images from healthy trees with other ones, this technology is able to identify the health status of urban trees [18,19] and forest trees [20]. Thus, it could distinguish healthy plants from the unhealthy ones. Work based on hyperspectral images have achieved good performance. However, analyses are sensitive to small changes in the surrounding environment, leading to a deterioration in the accuracy of the evaluation results. In addition, the constraint of this method is that only limited subjects can be evaluated simultaneously; thus, such an algorithm encounters difficulties when there are multiple subjects.

Considering the limitation of the aforementioned tree health assessment methods, we try to solve this problem in a novel manner. The internet of things has been an outstanding solution in various fields [21,22,23,24]. A distinguishing feature of the IoT network is that it can transmit real-time sensor-measured data to a backend server, which makes remote monitoring available. In general, an artificial intelligence (AI) algorithm is utilized to analyze collected data and make reasonable conclusions, making the whole process automatic [25,26]. The integration of an AI algorithm also improves the efficiency of applications. Motivated by this, a new method utilizing the IoT network and AI algorithm called the PTA algorithm is developed to assess tree health, which mitigates all the mentioned shortfalls. The objective of the proposed PTA is to provide early warnings of adverse health condition of trees by evaluating tree health. To achieve a sensible PTA, the key lies in how to reflect the surrounding environment. It is envisaged that PEFs, as proximity environmental features which are indicative of the tree health, should be identified for the development of the PTA algorithm. In this paper, seven PEFs are identified as the most important factors in photosynthesis and respiration among many environmental factors, which are essential blocking blocks for tree health growth. These PEFs are identified as air temperature, air humidity, oxygen concentration, carbon dioxide concentration, illumination intensity, soil humidity, and soil acidity in the PTA algorithm. Smart sensors are deployed to measure these PEFs. The collected data are transported through IoT networks and received by a server. A database composed of tree health (obtained by conducted visual tree assessments [5]) and corresponding PEF data is thus built in the server. The analytics algorithm at the backend server proliferates the evaluation of PTA. However, another problem existing is that lots of abnormal data are involved in the dataset. They are generated by various extrinsic factors such as weather changes. To solve this problem, an adaptive data identifying (ADI) algorithm is developed in this paper and applied to the database to exclude abnormal data. To find the relationship between the PEFs and tree health, the radial basis function (RBF) neural network (NN) has been identified as the appropriate tool in PTA algorithm. RBF NN is best suited for learning scenarios where the hidden relation is nonlinear [27] and the input dimension is sufficiently small [28]. It also has fast and accurate convergence. In this study, tree health states changed non-linearly according to seven PEFs (seven-dimension is considered as low dimensional). Thus, the RBF NN is adopted to model the PTA variations under different factor attributes. Finally, the developed PTA algorithm gives an evaluation result of the objective tree and provides important guidance to the community.

The key contributions of this paper are as follows:

1. A novel environmental factor-based tree health assessment method, the PTA algorithm, is developed to provide early warning for tree health conditions. Combined with environmental factors that can be measured by smart sensors, it can be utilized to monitor tree health remotely and automatically take advantage of the IoT network and AI algorithm.
2. To properly represent surrounding environments, seven main PEFs, are identified as essential factors in tree health evaluation. These seven factors account for the key impact on tree health due to changes in the surrounding environment comprehensively.
3. To alleviate the negative effects produced by interference factors on the PEF dataset, an ADI algorithm is developed. The results show that the ADI algorithm can effectively improve the performance of the evaluation model.

The rest of this paper is organized as follows. The PTA algorithm is described in detail in Section 2. The obtained results and discussion are presented in Section 3. The conclusion is presented in Section 4.

## 2. Materials and Methods

### 2.1. Overview

**Figure 1:**
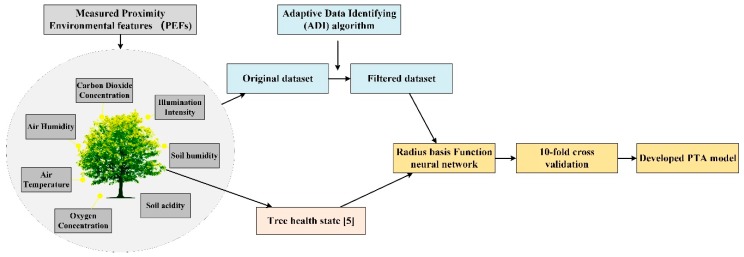
The PTA algorithm.

The proposed method follows the PTA algorithm, as shown in Figure 1. It consists of three main parts: the PEFs measurement, adaptive data identifying algorithm, RBF NN-based data analysis, and finally, the evaluation model. In addition to the measured PEF data, the tree health state is needed before the data is analyzed by RBF NN. In this paper, the tree health state is obtained through a widely used visual tree assessment method [5]. In this procedure, there are two tree health indicators, namely defoliation and discoloration. Each tree is evaluated in these two aspects and two percentage scores are given. The final health state depends on the combination of the two scores and is further divided into four classes (0–3). In general, a smaller number refers to a better tree health status. The final model reflects the potential relationship between actual tree health and PEFs. When new PEFs are given, a tree health status will be provided by the established model. In this section, the main three parts of PTA algorithm will be introduced. PEFs, referring to proximity ambient features, are described in part I. The measured PEFs are utilized as the input for the PTA algorithm. In order to achieve a good performance, an ADI algorithm is developed in part II. With the incorporation of the ADI algorithm, the negative effects caused by different factors can be alleviated from the original dataset. In part III, RBF NN is utilized to find the relationship between the selected PEFs and the tree health. The RBF NN is a good candidate to deal with nonlinear and low-dimensional mappings problems which are encountered in PTA scheme. In the training phase, a dependent variable Y (tree health) is predicted using a number of given M-dimensional independent measurements V (several groups of input data). The main prediction objective is to find the most probable value of Y for each value of V based on a finite set of measurements and their associated Y values. Thus, the RBF NN learns mapping from the input set including V to an output set including Y. The establishment of output set is achieved by [5]. Finally, a 10-fold cross validation is adopted to verify the reliability of the PTA algorithm.

### 2.2. Proximity Environmental Feature (PEF)

Main PEFs, referring to environmental factors that affect tree growth, have been taken into account in this study. These factors include air temperature, air humidity, oxygen concentration, carbon dioxide concentration, illumination intensity, soil humidity and soil humidity, as shown in Table 1. These seven environmental features, which are in a dynamic change, play the most important roles in tree growth. If one of these features is not in the normal range, trees cannot survive. Other factors are not involved in this algorithm because of the slight influence of, or being similar to, the mentioned PEFs. For instance, wind exposure contributes to tree health, but the influence is trivial, and thus, it is not included here.

Air Temperature (AT): air temperature is measured by a $0.6 digital temperature and humidity sensor DHT11 Temperature is a very important feature for the growth of trees because most chemical reactions which occur in trees are influenced significantly by temperature. Under unsuitable temperatures, trees cannot survive.

Air Humidity (AH): air humidity is synchronously measured by the digital temperature and humidity sensor DHT11. Water is the necessary raw material and catalyst for many chemical reactions. Too high humidity will cause the growth of micro-organisms and bacteria that harm trees. However, too low air humidity is not good because the leaves of trees will dry out according to the osmotic pressure.

Oxygen Concentration (OC): oxygen concentration is measured by a $20 oxygen sensor, O2A2. Oxygen is an essential environmental factor for all creatures on earth. Trees cannot live in an environment with too high or too low oxygen concentrations; both of these conditions prevent tree healthy growth.

Carbon Dioxide Concentration (CDC): carbon dioxide concentration is measured using a $12 carbon dioxide detection sensor MH-Z19B. Carbon dioxide is an important input for photosynthesis. If there is not enough carbon dioxide in the surrounding air, trees die because they cannot photosynthesize.

Illumination Intensity (II): illumination intensity is measured by a $2 illumination sensor, TSL2561. Light provides power and stimulate chlorophyll activity in the photosynthesis.

Soil Humidity (SH): soil humidity is measured by a $1 soil humidity sensor, FC-28. Different from water in air, water in soil is directly absorbed by trees and is engaged in photosynthesis. Too low humidity will slow down photosynthesis and cause trees to die of “thirst”. In contrast, too high humidity will break down healthy roots.

Soil Acidity (SA): soil acidity is measured by a $70 soil acidity sensor, KD21B20. Different pH values of soil can affect the availability of nutrients. If the pH of soil is too high or too low, the roots rot and trees may die.

All of these PEFs can be measured by smart sensors. The measured PEF will then be transmitted and recorded in a device for further analysis. The feature vector *V* with features $v_{n}$, for *n* = 1, 2, …, *M*, is formulated as Equation (1):

(1) $$V=\left[\begin{array}{cccc} v_{1} & v_{2} & \ldots & v_{M} \end{array}\right]$$

### 2.3. Adaptive Data Identifying Algorithm (ADI)

**Figure 2:**
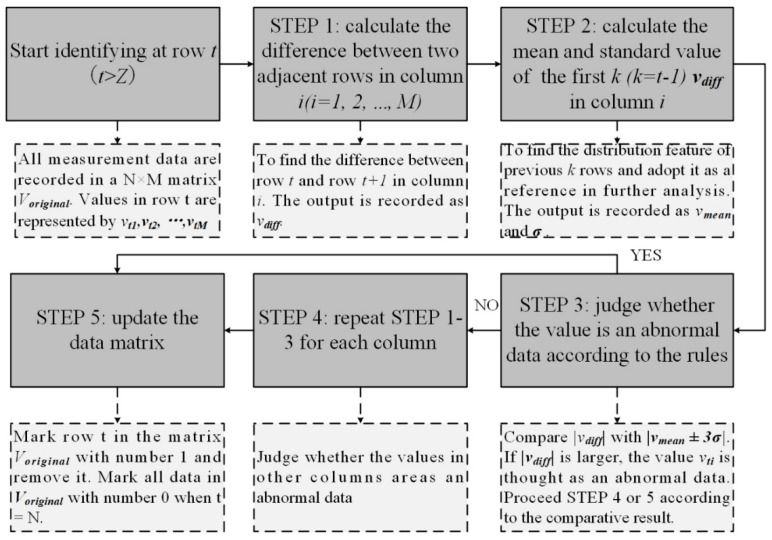
Data identifying algorithm.

After measurement of the PEFs, ADI algorithm is applied as a pre-processing method to alleviate the negative influence of emergencies in the test. These emergency factors may affect the ambient features. These factors change the values of the measured features and renders a deviation from the nominal values. The emergence of emergency factors is attributed to the that the period of data measurement is relatively long, and some unstable factors may influence the measured PEFs. Weather is considered a main factor because it can affect almost every PEF (e.g., the temperature suddenly drops a lot because of the coming of cold air. The illumination intensity may be influenced if it is rainy or cloudy rather than sunny) when it changes. In addition to the weather influence, wrongly recorded data (e.g., wrongly recorded because the control module is out of power) is another factor that will influence the performance of evaluation model. The influence of these kinds of factors is alleviated by the proposed ADI algorithm. Season is also an important factor that has a negative effect on the ambient features. However, the adverse effects caused by this factor will not be included in the raw data, because the period of all measurements does not span across two seasons. Figure 2 illustrates the procedures of the proposed adaptive filter algorithm.

In the ADI algorithm, we consider a sudden change in a value as abnormal. The difference between normal and abnormal values is large. If we put them in the training phase, the performance of training model will degrade. A simple solution is to remove data whose value are extreme. However, if we do so, some normal data may be also removed. It is known that the environment around a tree keeps changing in a suitable range. Although the value is at the edge of range, it may still belong to the normal class. Thus, we consider a change between two adjacent data in time series as the indicator. If the difference value is too large and not in line with the trend, the newly input data will be considered as abnormal.

At the beginning, an original dataset is formulated. The original dataset is formed as a data matrix, which is formulated using Equation (2):

(2) $$V_{original}=\left(\begin{array}{ccc} v_{11} & & v_{1M}\\ & \ddots & \\ v_{N1} & & v_{NM} \end{array}\right)$$

*N* represents the total number of the measured data vector *V* for one tree. As mentioned, there are *M* features in each vector. The matrix is formulated of *N* groups of measured data vector in a chronological order. In Step 1, the difference between the two adjacent rows is calculated and defined as Equation (3).

(3) $$v_{diff,i}^{t}=v_{i}{}^{t+1}-v_{i}{}^{t},\;t\;>\;Z$$

In Equation (3), *V* is a feature vector with *M* columns. The difference value in each column is calculated and recorded. *Z* represents the number of vectors which are recorded as the standard data in the dataset. These standard data reflect the normal distribution of difference and the newly recorded data will be compared with these standard data. If the newly recorded data do not submit to the distribution characteristic of standard data, it will be marked. A judge principle is made in ADI algorithm to make sure the abnormal data will be correctly recognized. The principle is formulated as follows:

If $\left|v_{diff,i}^{t}\right|>\left|v_{mean,i}^{t}\pm 3\sigma_{i}{}^{t}\right|$ the value is considered as abnormal data.

Each feature will increase from its minimum and achieve a maximum in one period (i.e., one day) Thus, the value of each feature in a period roughly submits to Gaussian distribution. In Gaussian distribution, 99.7 percent of all data are included in the range |μ±3σ|. Similarly, if there is a probability of 0.3 that the data belong to the distribution, it will be considered as abnormal data.

$v_{mean,i}^{t}$ and $\sigma^{t}$ are defined as the average value and standard value of the first (*t* − 1) difference values in column *i*, as is defined in Equations (4) and (5).

(4) $$v_{mean,i}^{t}=\frac{1}{t-1}\sum_{n=1}^{t-1}v_{diff,i}^{n}$$

(5) $$\sigma_{i}{}^{t}=\sqrt{\frac{\sum_{n=1}^{t-1}{\left(v_{diff,i}^{n}-v_{mean,i}^{n}\right)}^{2}}{t-1}}$$

If a $v_{diff,i}^{t}$ is considered to be abnormal data, the values of *t^th^* row matrix will be eliminated from $V_{original}$. $V_{mean,i}^{t}$ and $\sigma_{i}{}^{t}$ are calculated again with the new values. ADI algorithm will keep verifying the reliability of each group of data vectors in the *M* columns by continuously repeating the above process until the whole dataset is verified. If the any difference value in all columns is identified as abnormal, the whole vector will be removed from the original dataset.

With the ADI algorithm, all data that will confuse the training model and thus influence the performance are determined. However, they cannot be simply removed during training. Although produced by influence factors, these abnormal data are recorded by the deployed sensors. In the PTA algorithm, the whole system will be utilized for further tree health monitoring. The abnormal data will emerge again when there is similar interference. Thus, the recognized abnormal data will be masked by number 1. The number 0 is used to mask the normal data which are still in the original dataset. This number will be integrated into the feature vector in the (*M +* 1) column. The new feature vector is formulated in Equation (6).

(6) $$V_{new}=\left[\begin{array}{ccccc} v_{1} & v_{2} & \ldots & v_{M} & d \end{array}\right],\;d=0\;\mathrm{or}\;1$$

### 2.4. Radial Basis Function (RBF) Neural Network (NN)

The hidden relationship between tree health and the new feature vector (with *M* + 1 features) is explored by RBF NN. It is obvious that the value of tree health index changes in a nonlinear manner when different PEF attributes. For instance, if the temperature, one of the identified PEFs, is too high or too low, the tree will be in a poor health. It is well known that when the temperature is in a suitable range, the tree will grow in a healthy way. In addition, the dimensionality of the PEFs is seven - this is considered as a low dimensional number in data classification problems. Thus, it is considered as a nonlinear and low-dimensional classification problem, and a suitable classifier is needed. The RBF NN is a typical feedforward NN with the merits of simple structure, fast training capability, and capability of convergence to global optimization [27]. The basic idea is to transform the low-dimensional input vector into the high-dimensional space in the hidden layer; thus, unsolvable non-linear problems in low dimensional space can be solved in high dimension. The main advantage is that it can approximate arbitrary nonlinear functions with a high learning speed because of the simple structure [28]. Thus, by incorporating the non-linear and low-dimensional characteristics, RBF NN is adopted as the best solution to resolve such problems.

The relationship between input layer and output layer is formulated as Equation (7). It is a linear relationship between the input and output. The parameter σ and *c_i_* in Equation (8) represent the standard deviation and center point respectively. Parameter *w_ij_* represents the weighting of each Gaussian function.

(7) $$y_{j}=f_{j}(x)=\sum_{i=1}^{n}w_{ij}h_{i}(x),j=1,2,\ldots ,n$$

(8) $$h_{i}(x)=\exp(-\frac{1}{\sigma_{i}^{2}}||x-c_{i}|{|}^{2}),i=1,2,\ldots ,n$$

There are three parameters need to be identified when RBF NN is training: the center of base function, variance, and the weighting between hidden layer and output layer. These parameters were randomly initialized and then optimized by gradient descent.

In the training phase, it is necessary to decide when to stop the fitting process. Excessive training will delay the experiment and the performance of training model will not be improved. In order to balance the time cost and model performance, an error coefficient $E_{c}$ is defined as Equation (9). If $E_{c}$ is smaller than the threshold, training will stop.

(9) $$E_{c}=\sqrt{\frac{\sum_{k=1}^{k_{\max}}\sum_{i=1}^{i_{\max}}{\left(E_{d}^{ki}\right)}^{2}}{k\times i}}$$

In Equation (9), $E_{d}^{ki}$ represents the error distance, which is defined in Equation (10). The symbol ‖.‖ represents the abstract value of the returned real number.

(10) $$E_{d}^{ki}=\left\|Y_{predict}^{ki}-Y_{real}^{ki}\right\|$$

The performance of the proposed PTA algorithm is validated by 10-fold cross validation. A 10-fold validation is a conventional training and validating method in performance evaluation of classifier. The procedures are as follows:

Step 1: Divide all samples into ten groups randomly with an equal number of samples.

Step 2: Establish the classifier model using nine groups (90%) of samples.

Step 3: Validate the classifier model using the remaining one group (10%) of samples.

Step 4: Record the validation results (accuracy).

Step 5: Swap one group of samples in training set with the group of samples in validation set (the previous validation group should not be picked as validation data one more time)

## 3. Results

**Figure 3:**
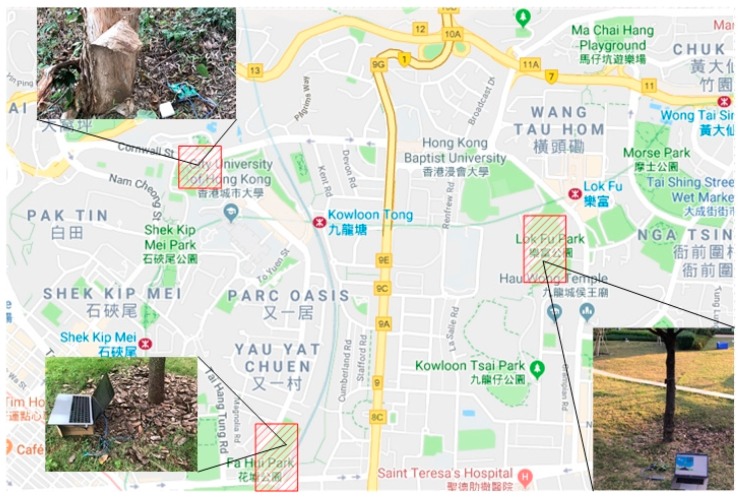
The distribution of test areas.

**Figure 4:**
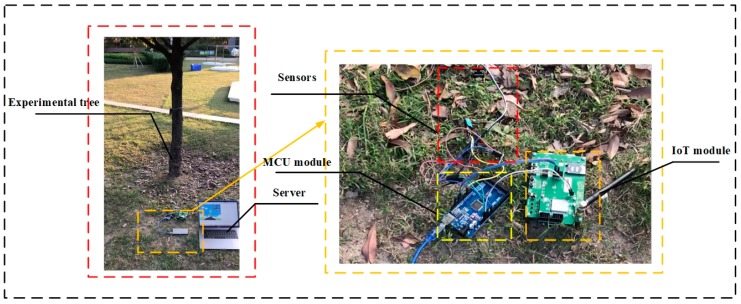
The experimental scene.

One hundred trees distributed in parks and campus were selected to verify the effectiveness of the proposed PTA algorithm. A map that describes the distribution of test areas is shown in Figure 3. The test areas were in the campus of City University of Hong Kong, Lok Fu Park, and Fa Hui Park. These trees were of the same type, i.e., camphor trees. In the experiment, a visual evaluation was conducted by five independent researchers following the guidelines described in [5]. The defoliation and discoloration of the leaves were estimated on a scale of 0–3. The final health score per tree was passed through the integration of two variables. The visual assessment was conducted once a day before the environmental features were measured. PEFs of objective tree were measured by the deployed acquisition equipment. The equipment consists of three parts, as shown in Figure 4, namely sensors, controller module and IoT module. The acquisition equipment was powered by the mobile power. In the experiments, sensors were deployed around the experimental object to measure ambient features. Each sensor was connected to a controller integrated module. The function of the module was to issue commands such as “decision when to collect data” and “how long will the collection be conducted”. etc. The module was then connected to the IoT module. Under the order of controller module, the collected data were transported to the server by the IoT network. A model based on PTA algorithm was established in the server. By accepting the transmitted data, the health states of experimental trees were predicted. The predictive results were compared with the previously evaluated tree health state. The experiments were conducted during the day time (10 a.m. to 4 p.m.) to avoid huge floating in the dataset, which would have a negative effect on the performance of the analyses. The time interval we set for the data collection was 10s. The measurements lasted for a few months. About 40,000 sets of data were gathered and utilized for further analysis. The results are shown in Table 2. The samples represented the data vectors collected from trees with relative health states. In the testing, most of trees were in great health or good health. To render the classification results reasonable, the number of samples of each health scale must be roughly similar. Thus, during testing, more data were collected from trees with moderate or poor health. Since the period of the experiment was long, the weather at the experimental sites changed from time to time. Sunny days were set as the reference of weather since most days when experiments were conducted were sunny. The ADI algorithm was thus adopted to remove abnormal data. After the incorporation of ADI, about 36,000 sets of data remained, and a dataset was built. RBF NN was then adopted to obtain the relationship between PEFs stored in the dataset and health state. Ten-fold cross validation was used in the training and validation phase. After 10 rounds, the mean value of accuracy was set as the final accuracy. To evaluate the complexity of proposed solution, the training time of each training was recorded. The test was conducted on a machine with Intel Core i5-3230M CPU @ 2.60 GHz and 2.60 GHz, and GPU NVDIA GeForce GT 750M. The average training time was calculated as 10.5 s.

**Figure 5:**
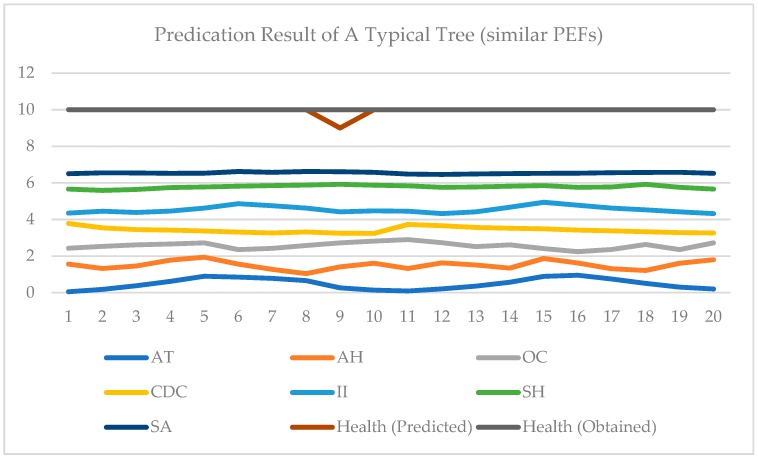
Prediction results of a typical tree (similar PEFs).

**Figure 6:**
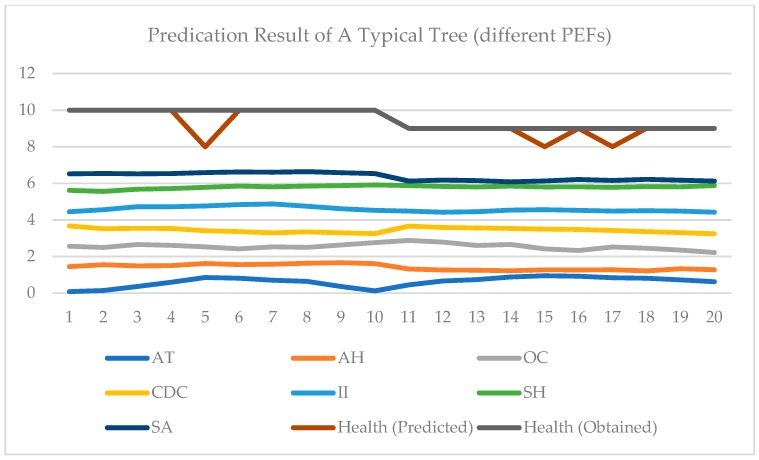
Prediction results of a typical tree (different PEFs).

**Figure 7:**
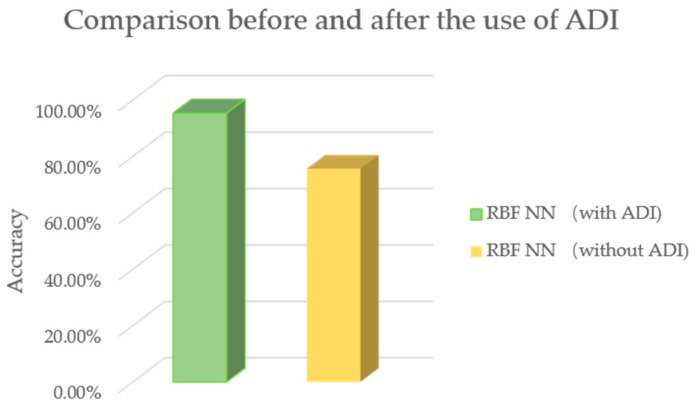
Comparisons results with or without ADI algorithm.

In Figure 5 and Figure 6, two examples of testing data are shown. In each figure, the test data and results of two days are given. All 7 PEFs data are normalized in [0, 1]. In Figure 5, the data of one typical tree for two days are displayed [0, 7]. A health score of [0, 3] is represented by [7, 10]. Data from two consequent days are included in Figure 5. It can be seen that since changes are limited, the health score does not vary accordingly. In contrast, in Figure 6, it may be observed that the PEFs of these two days are very different. These two days are extracted from one typical tree’s measurements, and they are not adjacent. It can be seen from Figure 6 that under the influence of changed environment factors, the health score changes. This proves the impact of PEFs on tree health. Figure 7 shows the effectiveness of the proposed ADI algorithm. Comparing to the accuracy of the established model without the ADI algorithm, the one with ADI algorithm increased by 19.7% The performance of the proposed PTA is evaluated in terms of average accuracy. If the evaluation result is same as the result obtained by a visual tree assessment, the result will be considered accurate. In this experiment, all classification schemes are evaluated under the same scenario, i.e., the same training data, and same testing data. Four (4) schemes are compared, namely: RBF NN, K-nearest neighbor (KNN), support vector machine (SVM) and recurrent neural network (RNN). KNN is a classifier based on the majority rule. In KNN, a hyper-parameter k is set for classification. For each class in a standard database, there are same numbers of samples. To determine the category of the new input, a Euclidean distance between the input and each sample in the standard database is calculated and sorted. In the first k smallest distances, the class of the largest sample becomes the new input. In our test, the k is set as 7. In SVM, the main parameters needed to be set are the type of kernel function and value of cost. In this experiment, the gaussian kernel is selected and the cost value is set as 0.001. When choosing the gaussian kernel, a special parameter gamma is set as 0.1 for gaussian kernel. A nonlinear autoregressive with external input (NARX) neural network, which belongs to RNN, is used in this paper. There are four parameters, namely input delays, feedback delays, hidden sizes, and train Fcn included in NARX neural network. The input delays and feedback delays represent the number of previous input and feedback that will be utilized to obtain the current output. The hidden sizes represent the number of sub-classifiers that set in the hidden layer. The final classifier is a combination of these sub-classifiers. The train Fcn represents the training function. In this experiment, input delays and feedback delays are both 16. The hidden size is 5, while the training function is train lm. In the experiments, a grid-search has been done, and it was shown that the selected parameters of all the methods were optimal.

**Figure 8:**
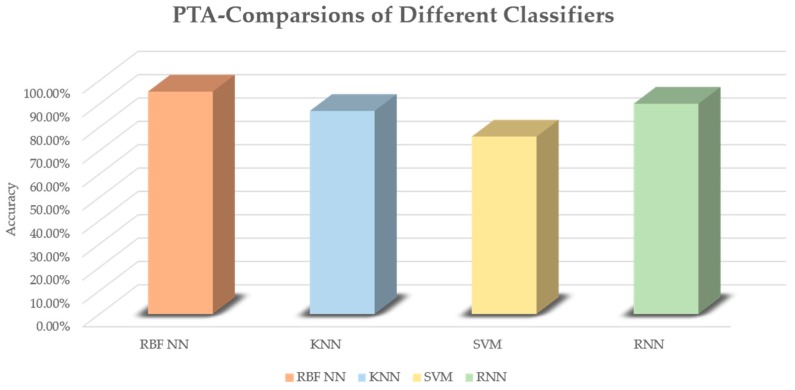
Evaluation of the proposed PTA (comparisons on different classifiers).

In the experiments, 10-fold validations were performed to acquire the results. An average accuracy is obtained as the final result of each classification. In these classifiers, corresponding parameters are adjusted to achieve the best performance of the classifier. The highest accuracy of each classifier trained model is recognized as the final result. As shown in Figure 8, the highest model accuracies of KNN, SVM and RNN were respectively 87.03%, 76.03% and 90.10%. The RBF NN achieved the best performance, with an accuracy of 95.3%. A confusion matrix is given in Table 3 to show the predicted accuracy in each class. T represents the true condition of sample classes while P represents the predicted condition of sample classes. The results show that class 3 has the best performance and class 2 the worst performance in the matrix. A comparison between the proposed algorithm and other related work is shown in Table 4. Two methods based on satellite data and two methods based on hyperspectral images are used to make the comparison. It is worth mentioning that our algorithm is applied for single tree evaluations, while the others concentrate on forest tree health evaluations. Thus, the results only show that our algorithm has advantages in some scenarios. In addition, an evaluation was conducted to verify the importance of each PEF. In the training, one of seven PEFs was removed and the evaluation result showed the importance of each value. The results are shown in Table 5. Though the importance of each PEF is different, each is necessary, since the accuracy decreases compared to the result when seven PEFs are involved.

## 4. Conclusions

Improperly grown trees cause huge hazards to the environment and to human society, so effective tree health monitoring methods are needed. However, traditional visual tree health assessment methods that ensure tree health cost a lot of labor and time. To provide monitoring priorities and manage valuable natural capital in a more efficient manner, a proximity environmental feature-based tree health assessment (PTA) algorithm has been developed to evaluate tree health based on the environmental factors described in this paper. In the development of the PTA algorithm, seven proximity environmental feature (PEFs) have been defined to describe the surrounding environment of trees. An adaptive data identifying (ADI) algorithm is developed to preprocess the gathered dataset to allow the evaluation model to achieve better performance. It is worth mentioning that PTA evaluates tree health based on sensor-measured ambient features; thus remote and automatic monitoring can be achieved by taking the advantage of IoT network and AI algorithm. In this investigation, only one type of tree has been evaluated in this paper, further studies will be undertaken for more samples of other types.

## Figures and Tables

**Table 1:** Selected proximity environmental feature.

Feature	Description
Air Temperature (AT)	The environmental temperature
Air Humidity (AH)	The content of water in the air surrounding the trees
Oxygen Concentration (OC)	The content of oxygen in the air surrounding the trees
Carbon Dioxide Concentration (CDC)	The content of carbon dioxide in the air surrounding the trees
Illumination intensity (II)	The intensity of light that leaves (or other parts) of trees could be received
Soil Humidity (SH)	The content of water in the soil surrounding the trees
Soil Acidity (SA)	The acidity of the soil surrounding the trees

**Table 2:** Health distribution of samples.

Health Scale	Performance	Number of Samples
0	Great	11306
1	Good	10421
2	General	10244
3	Poor	9302

**Table 3:** The confusion matrix.

Metric	Class 0 (T)	Class 1 (T)	Class 2 (T)	Class 3 (T)
Class 0 (P)	94.72%	4.58%	1.20%	0.11%
Class 1 (P)	3.21%	92.06%	3.19%	0.47%
Class 2 (P)	1.63%	2.46%	95.13%	0.77%
Class 3 (P)	0.44%	0.90	0.48%	98.75%

**Table 4:** The performance comparison between related work and proposed PTA algorithm.

Related Work	Overall Accuracy
Satellite data-based [14]	62%
Hyperspectral imaged-based [19]	81%
Satellite data-based [13]	88%
Hyperspectral imaged-based [18]	90%
Ours	95.3%

**Table 5:** The performance evaluation without each parameter.

Parameter	AT	AH	OC	CDC	II	SH	SA
accuracy	85.2%	87.6%	93.5%	82.2%	80.8%	88.2%	91.0%
